# Brief introduction to Synergy-COPD

**DOI:** 10.1186/1479-5876-12-S2-S1

**Published:** 2014-11-28

**Authors:** Adina Ratoi

**Affiliations:** 1 European Commission, DG CONNECT - H1 "Health & Well-Being", Brussels, Belgium

## 

Today the world is changing at a rapid pace and we are the witnesses of unprecedented technological advancements. In the health sector the range of ICT solutions and applications has been growing exponentially, nudging innovation, fuelling competition and stimulating economic growth.

Yet, none of these technological developments would be possible had there not been any societal pressing needs to address. Faced with demographic changes, increased prevalence of chronic diseases, rising healthcare costs and staff shortages, how could the EU provide affordable and quality services to its citizens? And more importantly, how could ICT's great potential be stimulated and exploited in the health sector, where paradoxically its penetration is still low? How could Europe enhance its competitiveness on the global economy? The answers to these questions and the potential solutions were to be addressed through specific objectives under the *7^th ^Framework Programme for Research and Technological Development *2007-2013 (FP7).

Overall, from a technological perspective, in the health sector the emphasis was supposed to be put on more accurate early diagnosis, disease prevention, and treatment, as well as ICT solutions and environments for integrative research. However, the core ICT research targets of FP7 were personal health systems, patient safety and the Virtual Physiological Human (VPH).

The concept of Virtual Physiological Human has seen a constant evolution since 1993 (the IUSP Conference) through 2006 (STEP project sponsored by the European Commission), leading towards the design of a VPH roadmap under FP7. The VPH framework was meant to facilitate the collaborative and integrative investigation of the human body/organs as a single complex system through the development of patient-specific computer models and simulators for applications in personalised and predictive medicine.

In 2010, under FP7 Call 6, the selected Virtual Physiological Human projects received roughly € 63 M in grants from the European Commission. SYNERGY-COPD (*Modelling and simulation environment for systems medicine - Chronic obstructive pulmonary disease - COPD - as a use case*) was one of the project to benefit from this funding.

In response to the call, a consortium made of 9 partners proposed an ambitious project aiming at addressing the theme of chronic obstructive pulmonary disease through computer modelling and simulation. COPD is a very complex disease, which represents a major health problem throughout the world causing chronic morbidity and mortality.

The project started in early 2011 under the coordination of Mr Felip Miralles from Barcelona Digital, an ICT technology centre in Spain. In this endeavour, BDIGITAL has been backed by 9 other partners from 5 different countries, all with a strong expertise in the medical research field, from academia (Karolinska Institute, the Chancellor, Masters ad Scholars of the University of Oxford, the Technical University of Budapest, the University of Liverpool continuing the work of University of Birmingham) to clinical research organisations (Linkcare Health Services and IDIBAPS) and software companies (Biomax AG and Infermed). Such a multi-disciplinary and multi-organisational approach corroborated with the know-how in path modelling played a key role in the attempt to achieve the objectives defined in the project proposal.

What the SYNERGY-COPD project proposed was to develop a decision-support system and a simulation environment which would enable the deployment of systems medicine to be applied to chronic diseases based on the following elements: a knowledge base, a simulation engine and two graphical visualisation environments. Moreover, in order to demonstrate the impact of the project on the medical research and clinical practice, the use case scenarios was a valuable component. As such, the system was planned to be modelled and validated through datasets from real patients affected by COPD. Concretely, the main goal of the project is to develop subject-specific computational models of fluid transport (air and blood) within the respiratory system, while outputs are to be integrated into a multi-scale trans-European model of oxygen delivery in patients with chronic obstructive pulmonary disease.

As it may be noticed from above, the SYNERGY-COPD proposal engaged to address the target defined in the call for proposals, i.e. to develop a ***patient-specific computer based model and simulation****[of the physiology of human organs and pathologies] *by integrating data from different disciplines into biological models via mathematics, computer science and engineering so as to provide a holistic understanding of the COPD for both physicians and bio-researchers. In the first 2 years since it started, the project proved to have made good progress in achieving the goals set, as it will be explained in the following pages.

Also, in order to demonstrate its European dimension and added value, the SYNERGY-COPD project - with a European team on board - took on to develop a solution that would position the European industry of medical simulation, decision support, and integrated healthcare in a better spot.

There is no doubt the project is very clinically oriented and that it has great potential to produce significant scientific impact. Already in its 3^rd ^and final year, expectations are set high and results are being eagerly awaited.

## Competing interests

Adina Ratoi is a Project Officer at the European Commission, Directorate General for Communications Networks, Content and Technology, the co-funding body for the Synergy-COPD project.

## Declaration

Publication of this article has been funded by the Synergy-COPD European project (FP7-ICT-270086). The article was written in January 2014; at that time the project was not completed yet.

This article has been published as part of *Journal of Translational Medicine *Volume 12 Supplement 2, 2014: Systems medicine in chronic diseases: COPD as a use case. The full contents of the supplement are available online at http://www.translational-medicine.com/supplements/12/S2.

